# Correlation-driven electronic nematicity in the Dirac semimetal BaNiS_2_

**DOI:** 10.1073/pnas.2212730119

**Published:** 2022-12-02

**Authors:** Christopher John Butler, Yuhki Kohsaka, Youichi Yamakawa, Mohammad Saeed Bahramy, Seiichiro Onari, Hiroshi Kontani, Tetsuo Hanaguri, Shinichi Shamoto

**Affiliations:** ^a^RIKEN Center for Emergent Matter Science, Wako, Saitama 351-0198, Japan; ^b^Department of Physics, Nagoya University, Nagoya 464-8602, Japan; ^c^Department of Physics & Astronomy, University of Manchester, Manchester M13 9PL, United Kingdom; ^d^Neutron Science and Technology Center, Comprehensive Research Organization for Science and Society, Tokai, Ibaraki 319-1106, Japan; ^e^Department of Physics, National Cheng Kung University, Tainan, Taiwan 70101, Republic of China

**Keywords:** correlated electron systems, electronic nematicity, Dirac semimetals

## Abstract

In BaNiS_2_, a Dirac nodal line band structure exists within a two-dimensional Ni square lattice system, in which significant electronic correlation effects are anticipated. Using scanning tunneling microscopy (STM), we discover signs of correlated-electron behavior, namely electronic nematicity appearing as a pair of *C*_2_-symmetry striped patterns in the local density-of-states at ∼60 meV above the Fermi energy. In observations of quasiparticle interference, as well as identifying scattering between Dirac cones, we find that the striped patterns in real space stem from a lifting of degeneracy among electron pockets at the Brillouin zone boundary. We infer a momentum-dependent energy shift with *d*-form factor, which we model numerically within a density wave (DW) equation framework that considers spin-fluctuation-driven nematicity. This suggests an unusual mechanism driving the nematic instability, stemming from only a small perturbation to the Fermi surface, in a system with very low density of states at the Fermi energy. The Dirac points lie at nodes of the *d*-form factor and are almost unaffected by it. These results highlight BaNiS_2_ as a unique material in which Dirac electrons and symmetry-breaking electronic correlations coexist.

## Significance

Topological electronic properties, usually understood in an uncorrelated electron picture, can be guaranteed by crystal symmetries. However, a material’s electrons can spontaneously form a pattern less symmetrical than the underlying crystal due to their mutual correlations, a complex problem underpinning phenomena such as high-temperature superconductivity. In the topological semimetal BaNiS_2_, we use atomically resolved microscopy to discover a spontaneous rotational symmetry breaking, or “nematic,” electronic phase. We elucidate the unusual mechanism by which this state emerges through electronic correlations and how the topological band crossings of the semimetal persist within this symmetry-broken environment. This helps to elucidate the interplay between topology and electronic correlations, foundational but often discordant concepts for quantum materials.

Materials in which Dirac fermions and significant electronic correlations coexist represent an intersection of two active frontiers of condensed matter research and might host a range of useful or interesting new phenomena (1, 2, 3). Correlations between electrons can result in instability toward a number of spontaneous symmetry-breaking configurations including magnetic order, charge density waves, and unconventional superconductivity. They can also lead to nematic order, which plays an important role in the cuprate and Fe-based superconductors (4, 5, 6, 7). Basic questions include whether topological band structures can survive in the presence of significant electronic correlations and whether, or in what way, the topological classification of a system can be altered by correlation-driven symmetry-breaking effects such as nematicity, in which translational symmetries are preserved while rotational symmetry is broken (8, 9, 10). However, the impact of such symmetry-breaking effects on Dirac fermions remains to be fully elucidated.

Correlations can be introduced through the inclusion of transition metals in the material composition, while otherwise ensuring the requisite crystal symmetries for an interesting topological phase (11). Such a situation is realized in the BaCo_1 − *x*_Ni_*x*_S_2_ series of compounds. It has recently been shown that the end member of the series, BaNiS_2_, hosts four simple Dirac nodal lines that are well isolated from other bands and traverse the Brillouin zone (BZ) with fairly weak dispersion along the layer-perpendicular axis (12, 13). These properties make it one of the systems closest to the ideal of a two-dimensional (2-D) Dirac fermion system. The bands emanating from these nodal lines have been shown to be susceptible to renormalization under ultrafast light pulses (14) and to a controlled shifting of the Dirac node energy and wavevector upon Co substitution (13).

The *d* orbitals of the transition metal square net in BaCo_1 − *x*_Ni_*x*_S_2_ introduce correlated-electron behavior, most obviously magnetism, but also further interesting properties. While BaNiS_2_ is a paramagnetic metal, BaCoS_2_ is an antiferromagnetic insulator (17). In the BaCo_1 − *x*_Ni_*x*_S_2_ solid solution, at *x* = 0.22 a metal–insulator transition occurs, for which both purely electronic (18, 19, 20) and structural mechanisms (21) have been proposed. Although superconductivity is absent, the temperature-composition phase diagram otherwise resembles that of the layered cuprates, and the above properties make BaCo_1 − *x*_Ni_*x*_S_2_ an attractive system for the exploration of possible composition-tuned Mott–Hubbard physics on a square lattice and the search for correlation-driven symmetry breaking phenomena. Even the metallic state of pure BaNiS_2_, furthest from the metal–insulator transition, has been shown to be somewhat anomalous, with *T*-linear resistivity below *T* = 2 K possibly hinting at the presence in the phase diagram of a quantum critical point (22).

In this work, we use scanning tunneling microscopy (STM) to explore the electronic structure at the cleaved surface of BaNiS_2_, observing both nearly ideal topological Dirac fermionic behavior and clear symmetry breaking in the form of bond-order nematicity within the surface Ni square net. In a narrow energy range approximately 60 meV above *E*_F_, we observe a lowering of symmetry in the surface local density-of-states from the expected *C*_4_ symmetry to two perpendicular striped patterns, each of *C*_2_ symmetry, and separated by an energy of ∼12 meV. From a corresponding lowering of the symmetry in quasiparticle interference (QPI) patterns, we infer a momentum-dependent energy shift of *d*-form factor. The Dirac points are located at the nodes of this form factor and are therefore left unaltered. Finally, we suggest a mechanism, modeled in the density wave (DW) equation framework, of interfering spin fluctuations as the driver of the nematic order.

## Properties of the BaNiS_2_(001) Surface

**Fig. 1.**
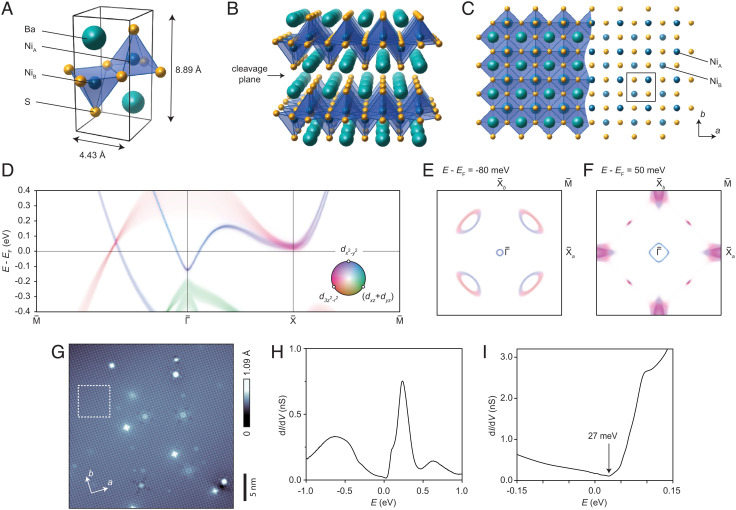
Overview of the crystal and low-energy electronic structures of BaNiS_2_. (*A*) Depiction of the primitive cell (15) and (*B*) quasi-two-dimensional crystal structure, showing the location of the cleavage plane. (*C*) Top-down view of the surface lattice. The cutaway view shows the Ni_A_ and Ni_B_ square nets. The primitive cell is shown with a black square. Unit-cell and lattice images were created using VESTA (16). (*D*) Numerically computed surface band structure shown along high-symmetry lines. The orbital composition for four of the Ni *d* orbitals is indicated according to the color key, with the radial axis (lightness) signifying the spectral weight summing over all orbitals. The *d*_*xy*_ orbital is not shown as its spectral weight is negligible in this energy range. (*E*) A constant-energy cross-section through *A*(**k**, *E*) at *E* = −80 meV and (*F*) a cross-section at *E* = 50 meV. In these calculations, the *P*4/*nmm* space group is assumed, resulting in *C*_4_ symmetry of the constant-energy cross-sections, but this assumption will be overturned by the observations of nematicity shown below. In anticipation of this, we draw a distinction between the points ${\bar{X}}_{a}$ and ${\bar{X}}_{b}$ at the zone boundary. (*G*) A typical constant-current STM topograph (setpoints *V* = 0.1 V, *I* = 100 pA). The observed atomic corrugations likely correspond to the uppermost Ni_A_ square net. (*H*) A $\frac{dI}{dV}\left(E\right)$ conductance curve after averaging over the field of view marked as a white dashed square in (*G*). (*I*) A $\frac{dI}{dV}\left(E\right)$ curve averaged over the same area, focusing on the region nearer to the Fermi energy *E*_F_.

BaNiS_2_ is a layered quasi-2-D material with a structure belonging to the nonsymmorphic space group *P*4/*nmm*, with lattice parameters reported as *a* = *b* = 4.43 Å and *c* = 8.89 Å (15). Its structure is composed of NiS_5_ pyramids whose orientation alternates about the basal plane and whose basal edges are shared with their neighbors, as depicted in Fig. 1*A*. Sandwiching these layers are Ba atoms located between, and almost in the plane of, the apical S of the NiS_5_ pyramids. As indicated in Fig. 1*B*, cleavage can occur between adjacent BaS layers revealing (001) facets. A top-down view of the surface lattice is shown in Fig. 1*C*. The cutaway on the right-hand side of the image shows the Ni_A_ and Ni_B_ lattices.

Fig. 1*D* shows the numerically computed (001) surface spectral function *A*(**k**, *E*) along chosen high-symmetry axes of the BZ. The orbital composition of the bands is shown using hue as indicated in the inset. Approximately halfway along the $\bar{\Gamma M}$ line, there exists a Dirac cone. This feature represents the surface projection of a 3-D cone emanating from one of the four Dirac nodal lines running between the $k_{z}=\frac{\pi }{c}$ and $-\frac{\pi }{c}$ planes (13). The electron-like pocket surrounding the $\bar{\Gamma }$-point results from a feature previously described as a pinched electron-like tube connecting the $k_{z}=\pm \frac{\pi }{c}$ faces of the BZ (23). A third noteworthy feature is the electron-like band centered around the $\bar{X}$ point. This results from the collapse into two dimensions of a band surrounding each of the R points at the edges of the bulk BZ, and the previously described Rashba splitting within these bands is also observed here (24, 25).

In Fig. 1*E*, we show a constant-energy cross-section taken from *A*(**k**, *E*) at *E* = −80 meV. Here, the electron-like pocket around the bulk *Γ* point, and hole pockets stemming from the Dirac nodal lines, are seen to collapse into a circle and a set of ellipses, respectively, in the surface BZ. The Dirac cones are composed of hybridization of the Ni *d*_*x*^2^ − *y*^2^_ and *d*_*z*^2^_ orbitals. Similar orbital hybridization has previously been observed or predicted at Dirac crossings in certain cuprate, nickelate, and Fe-based materials (26, 27, 28). Below the Dirac points, these orbitals contribute the inner ($\bar{\Gamma }$-facing) and outer ($\bar{M}$-facing) arcs, respectively (13), and this is reversed above the Dirac points as seen at the left-hand side of Fig. 1*D*. Fig. 1*F* shows a constant-energy cross-section at *E* = 50 meV, an energy which coincides closely with both the Dirac nodes and the bottom of the electron bands around the $\bar{X}$ points. Like the Dirac cones, these bands are composed of a hybridization of *d*_*x*^2^ − *y*^2^_ and *d*_*z*^2^_ orbitals. We point out that these numerical calculations do not accurately reflect the energies of these features, which are to be properly established through observations presented below (and to some extent in *SI Appendix*).

A typical constant-current STM topography image acquired at a cleaved surface is shown in Fig. 1*G*. The observed atomic corrugations almost certainly represent the Ni square net and specifically Ni_A_. (The periodicity of the corrugations corresponds to one observed atom per surface unit cell; *SI Appendix*.)

Fig. 1 *H* and *I* show typical tunneling conductance curves, denoted by $\frac{dI}{dV}\left(E\right)$, where, by convention, *E* = *eV* (*e* being the electron charge and *V* the sample bias). These are averaged over the 6 × 6 nm^2^ field of view marked as a white dashed square in Fig. 1*G*. The curves indicate a density-of-states close to an ideal semimetal, i.e., becoming small near the Fermi energy. In Fig. 1*I*, the “v”-shaped minimum at *E*≈ 27 meV can be taken to indicate the approximate energy of the Dirac crossings. The shallow and roughly linear onset toward lower energy likely corresponds to Dirac cones emerging from these crossings. Additional spectroscopy measurements acquired under magnetic fields are shown in *SI Appendix*. These indicate the emergence of a field-independent Landau level, indicating Berry’s phase of *π* associated with each Dirac point as well as giving a more precise estimate of the Dirac points’ energy. We note that *E*_F_ is located near the Dirac point energy, where the density-of-states is minimal. This situation would generally be unfavorable for electronic instability. However, as we will show below, BaNiS_2_ exhibits a likely correlation-driven electronic nematicity.

## Observation of Nematic Order

**Fig. 2.**
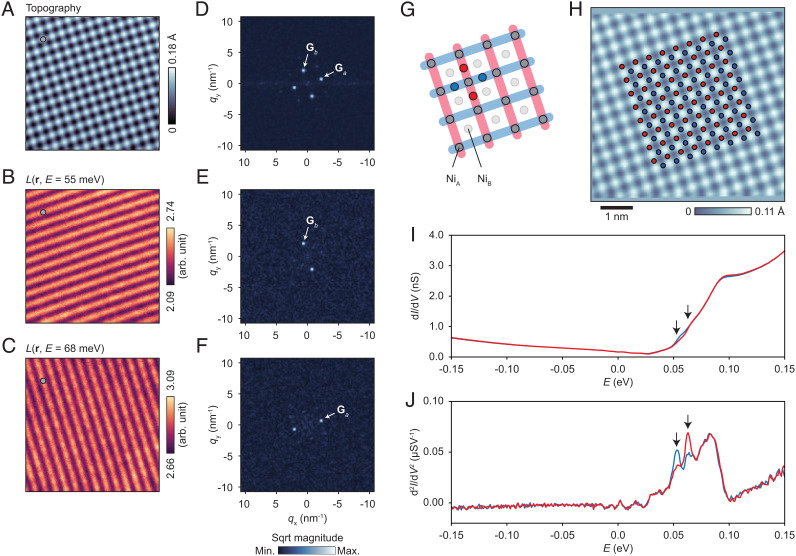
High-resolution spectroscopic imaging. (*A*) Constant-current topography in the 6 × 6 nm^2^ field of view marked by the dashed square in Fig. 1*G* (setpoints *V* = 0.1 V, *I* = 500 pA). (*B* and *C*) *L* images at selected energies (*V*_mod_ = 1 mV). The gray dots in (*A*–*C*) mark a single Ni_A_ site and show that the lines of high intensity in the *L* images coincide exactly with the Ni_A_ lattice. (*D*–*F*) Fourier transformations of the images in (*A*–*C*). (*G*) A depiction of the Ni_A_ square net and its two bond-centered sublattices, marked by arrays of blue and red dots. (*H*) Sampling of $\frac{dI}{dV}\left(E\right)$ data in the same field-of-view as in (*A*) (setpoints *V* = 0.15 V, *I* = 300 pA, and *V*_mod_ = 2.5 mV), on a 8 × 8 grid on each bond-centered sublattice. An affine transformation was applied to the data to coerce the atomic corrugations onto a square lattice to facilitate the sampling. (*I*) $\frac{dI}{dV}\left(E\right)$ curves after averaging over each of the sets of sampling points. Each arrow marks a subtle kink in the respective curve. (*J*) Derivatives $\frac{d^{2}I}{dV^{2}}\left(E\right)$ enhancing the small variations of the curves shown in (*I*). The splitting between the peaks is ≈12 meV.

Fig. 2 shows high-resolution spectroscopic imaging that reveals an unexpected symmetry breaking within the local density-of-states. Fig. 2*A* shows STM topography over the same 6 × 6 nm^2^ field of view as marked by a white dashed square in Fig. 1*G*. A gray dot in the upper left-hand corner marks one of the Ni_A_ sites for a point of reference. Differential conductance $\frac{dI}{dV}\left(r,E\right)$ was acquired by measuring a $\frac{dI}{dV}\left(E\right)$ curve at each pixel. Fig. 2 *B* and *C* show images extracted from the normalized differential conductance defined as $L\left(r,E\right)\equiv \frac{dI}{dV}\left(r,E\right)/\frac{I(r,E)}{V}$, at *E* = 55 meV and 68 meV, respectively. The normalization mitigates the so-called “set-point effect” (29), an unwanted influence of variations in tip-sample spacing on $\frac{dI}{dV}\left(r\right)$ images. The gray dots in Fig. 2 *B* and *C* are carried over from the one in Fig. 2*A*. The Fourier transform images of the topographic and *L*(**r**) images are shown in Fig. 2 *D*–*F*. In Fig. 2*D*, the peaks corresponding to the reciprocal lattice vectors **G**_*a*_ and **G**_*b*_ are seen and show the expected *C*_4_ symmetry. In Fig. 2 *B* and *C*, we see the two *C*_2_ symmetry stripe patterns, each commensurate with, and in phase with, one of the surface lattice corrugations [i.e., for each pattern **q**_*a*, *b*_ = **G**_*a*, *b*_ as seen in Fig. 2 *E* and *F*].

The peaks in the intensity of each striped pattern correspond directly with the rows or columns of the Ni_A_ lattice, and each stripe lies along a Ni_A_–Ni_A_ bond chain (or more strictly, a Ni_A_–S–Ni_A_ bond chain). This is reminiscent of the oxygen-centered nematic patterns observed in the cuprate high-*T*_*c*_ superconductors (4, 29). Fig. 2*G* shows a cartoon of this situation, in which the blue (red) stripes indicate the pattern at the lower (upper) energy. As well as the in-plane projections of the Ni_A_ and Ni_B_ sites, the sites belonging to the two inequivalent bond-centered lattices are also shown, with red and blue dots. As shown in Fig. 2*H*, we perform a sampling of the raw conductance data in the same field of view as Fig. 2 *A*–*C*, on a 8×8 grid on each of the sublattices. Fig. 2*I* shows the resulting $\frac{dI}{dV}\left(E\right)$ curves (averaged over the 64 sites of each grid). In each $\frac{dI}{dV}\left(E\right)$ curve, a subtle shoulder in the steep onset of states above the conductance minimum is marked with a black arrow. A derivative with respect to energy, shown in Fig. 2*J*, helps to clarify the difference between the two curves. A peak in the second derivative of an *I* − *V* curve is generally known to correspond to the availability of an additional tunneling channel, such as a band edge that becomes available upon reaching the necessary bias. The energy difference between the peaks in the two curves is ∼12 meV. In *SI Appendix*, we show the energy-dependent intensities of the Bragg peaks in the Fourier-transformed data (Fig. 2 *E* and *F*), for comparison with the curves shown in Fig. 2 *I* and *J*.

A question that reasonably follows from the above observation is whether a domain structure of the nematic order exists. Throughout this work, examining patterns of nematic order in two samples and searching over fields-of-view up to ∼500 nm, no domain boundaries were observed, and the order was seen to be preserved across a step-terrace morphology including three atomic terraces (*SI Appendix*).

## Manifestation of Nematicity in QPI

The mechanism underlying the observed nematic order might be elucidated by examining the momentum–space electronic structure at the BaNiS_2_ surface, and indirect insights into this can be obtained through interpretations of QPI phenomena. Such measurements can complement previous angle-resolved photo-emission spectroscopy reports (13, 14, 24) but can additionally probe unoccupied bands.

**Fig. 3.**
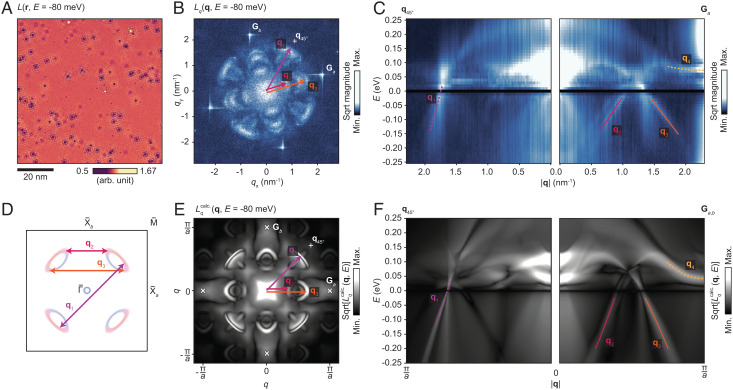
Overview of QPI observations. (*A*) A normalized conductance image *L*(**r**) acquired at *E* = −80 meV in a 80 × 80 nm^2^ field of view, showing QPI modulations around point defects (448 × 448 pixels, setpoints *V* = 0.25 V, *I* = 1 nA, and *V*_mod_ = 10 mV). (*B*) A Fourier transform of the *L* map, labeling the reciprocal lattice vectors **G**_*a*_ and **G**_*b*_. The wavevectors **q**_1, 2, 3_ of QPI modulations discussed in the text are marked with purple, red, and orange arrows. The point **q**_45°_ marks the endpoint of a vector formed by rotating **G**_*a*_ by 45°. (*C*) Linecuts through the *L*_*q*_(**q**, *E*) data, from the origin to **G**_*a*_ and also to **q**_45°_. The intensity is averaged over the line width of 10 pixels. Here, we additionally highlight the wavevector **q**_4_, which will be discussed below. The dashed lines for **q**_1_ and **q**_4_ are guides to the eye. The solid lines for **q**_2_ and **q**_3_ are obtained by a fitting procedure described in detail in *SI Appendix*. (*D*) A schematic of the surface BZ and calculated spectral function at *E* = −80 meV showing the likely scattering transitions giving rise to **q**_1, 2, 3_. (*E*) Calculated QPI pattern $L_{q}^{\mathrm{calc}.}\left(q\right)$ at *E* = −80 meV, based on the spectral function and *T*-matrix formalism (see *Methods*). Each white × symbol marks the position of a reciprocal lattice point. (*F*) Profiles through $L_{q}^{\mathrm{calc}.}\left(q,E\right)$ highlighting the predicted signals $q_{1,2,3,4}^{\mathrm{calc}.}$. The solid red lines mark the ridges in $L_{q}^{\mathrm{calc}.}\left(q,E\right)$, corresponding to **q**_2_ and **q**_3_, for comparison with the solid lines in (*C*).

Fig. 3*A* shows a selected image from *L*(**r**, *E*) data acquired in a larger field-of-view, at E=−80meV, in which modulations resulting from interference caused by scattering of quasiparticles from point defects are seen. Fig. 3*B* shows the Fourier transform F[L(r,E=−80meV)]. We hereafter refer to such Fourier-transformed conductance data loosely as *L*_*q*_(**q**, *E*). The reciprocal lattice vectors are again denoted by **G**_*a*_ and **G**_*b*_. At this energy, we identify three prominent scattering vectors, labeled **q**_1, 2, 3_. Fig. 3*C* shows linecuts through *L*_*q*_(**q**, *E*), running from the origin to **G**_*a*_ and along a line of the same length running halfway between **G**_*a*_ and **G**_*b*_. The most interesting features are seen in the linecut running from the origin to **G**_*a*_. The branches **q**_2_ and **q**_3_ form an inverted “v” below *E*_F_, and it can be inferred that they represent the shortest and longest possible scattering vectors, respectively, connecting two Dirac cones in adjacent quadrants of the BZ. At positive energy, a branch with apparently very weak dispersion, **q**_4_, extends to meet **G**_*a*_. This branch will be discussed in greater detail below.

Fig. 3*D* shows the likely origin of each of the scattering signals found at *E* = −80 meV. We attribute **q**_1_ to scattering between Dirac cones on opposite sides of the BZ. We attribute **q**_2_ and **q**_3_ to scattering between cones in adjacent quadrants of the BZ and specifically between the least- and most-distance ends of the respective ellipsoids as illustrated in Fig. 3*D*. Fig. 3 *E* and *F* shows the corresponding calculated QPI pattern and linecuts. All of the labeled QPI branches are qualitatively reproduced.

Comparing the data shown in Fig. 3 *C* and *F*, we find that the observed dispersions for **q**_2_ and **q**_3_ are only a factor of 0.58 as high as the calculated ones. With reference to recent related work, we speculate that the reason could be a failure to fully account for electronic correlations that might renormalize the band dispersion at low energies (30). Therefore, the inferred “squeezing” of the band structure near *E*_F_ may reflect significant electronic correlation effects in the real material.

A numerical fitting to the **q**_2_ and **q**_3_ branches, shown as red and orange solid lines in Fig. 3*C*, yields apparent velocities *v*_**q**_2__ = 8.15×10^4^ m s^−1^ and *v*_**q**_3__ = −6.91×10^4^ m s^−1^, respectively. However, these values are related only indirectly to any actual band velocities since a Fourier transform of QPI modulations visualized the momentum-transfer (*q*) space rather than the momentum (*k*) space. By making a comparison between the observed and calculated features in *q*-space, and also by making a *k*-space-to-*q*-space comparison of the calculation results, we can eventually estimate some key properties of the Dirac cones (full arguments in *SI Appendix*). In this way, the band velocities of the inner ($\bar{\Gamma }$-facing) and outer ($\bar{M}$-facing) sides of each Dirac cone are estimated to be *v*_*k*, inner_ = 3.09×10^5^ m s^−1^ and *v*_*k*, outer_ = −2.98×10^5^ m s^−1^, respectively. This is reasonably consistent with a rough estimate drawn from recently reported angle-resolved photoemission spectroscopy results, of |*v*_*k*, inner_|∼|*v*_*k*, outer_|≈ 2 × 10^5^ m s^−1^ (13).

**Fig. 4.**
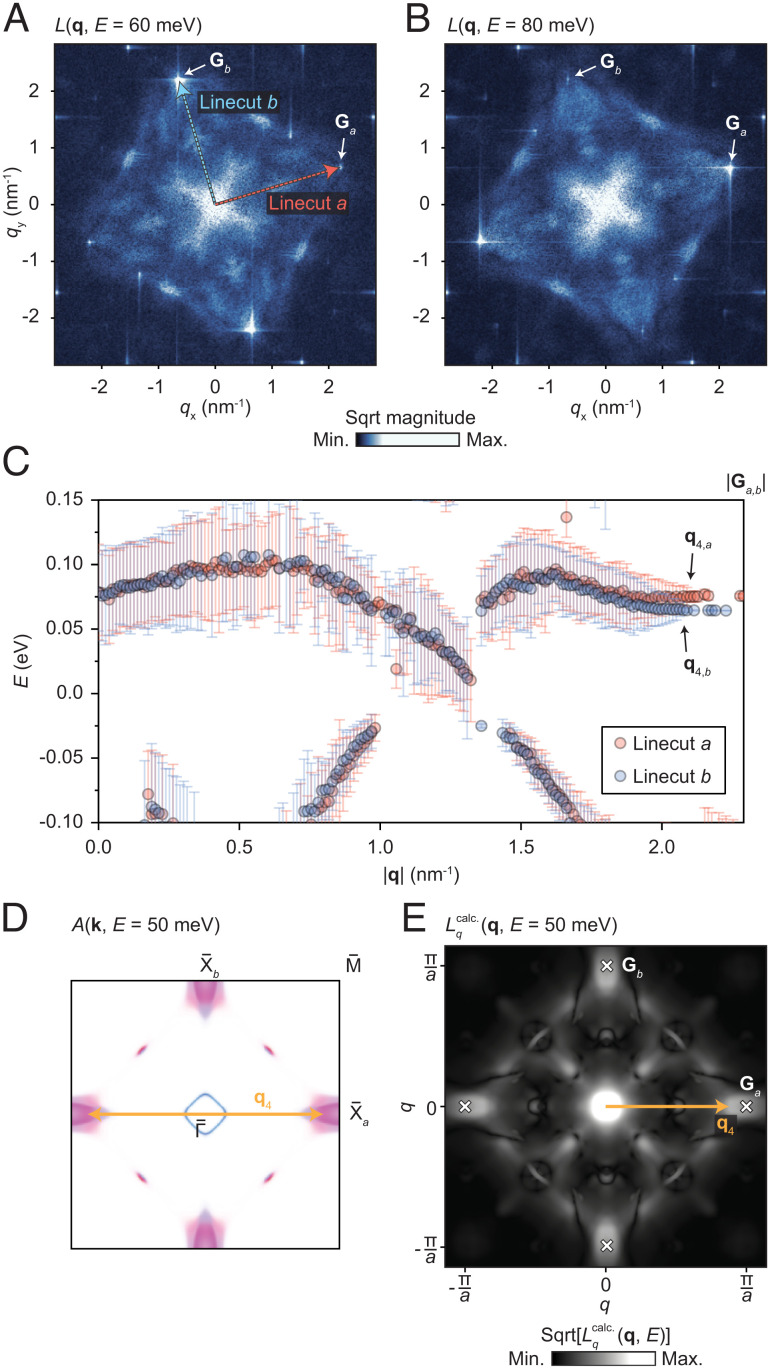
Symmetry breaking in QPI patterns. (*A*) Image $L_{q}\left(q,E=60\;\mathrm{meV}\right)$ from the same measurement described for Fig. 3 above. The pairs of reciprocal lattice peaks **G**_*a*_ and **G**_*b*_ show a large difference in intensity. (*B*) Corresponding image at *E* = 80 meV. Here, the alternate set of reciprocal lattice peaks becomes more intense. (*C*) Comparison of fitting to *L*_*q*_(*q*, *E*) along the **G**_*a*_ and **G**_*b*_ vectors [i.e., along the red and blue dashed lines marked in (*A*)]. In the region in which *q* approaches |**G**_*a*, *b*_|, the branches of points diverge, toward a splitting of ∼12 meV at the endpoint. (*D*) Schematic of the surface BZ and calculated spectral function at *E* = 50 meV, with the yellow arrow illustrating the scattering vector *q*_4_. (*E*) Corresponding calculated QPI pattern $L_{q}^{\mathrm{calc}.}\left(q\right)$ at *E* = 50 meV. See also Fig. 3*F*.

Having identified the *q*-space signatures of the Dirac cones, we turn our attention to the manifestation in QPI phenomena of the nematic behavior described above. Examining the *L*_*q*_(**q**, *E*) data further, we find that in a small energy range a few tens of meV above *E*_F_, there appears a lowering of symmetry similar to that shown in Fig. 2. Two *L*_*q*_(**q**) images selected from the same data as shown in Fig. 3, at *E* = 60 meV and *E* = 80 meV, are shown in Fig. 4 *A* and *B*, respectively. Each image shows an asymmetry in intensity between the pairs of reciprocal lattice peaks **G**_*a*_ and **G**_*b*_ [recall Fig. 2 *E* and *F*].

Fig. 4*C* shows the result of fitting a sum of multiple Lorentzian functions to the *L*_*q*_(*E*)|_*q*_ curve at each *q*-point along the lines from **q** = 0 to each of the reciprocal points **G**_*a*_ and **G**_*b*_. (This is the precursor step to obtaining the linear fits shown in Fig. 3*C*, and the full details are described in *SI Appendix*). The results from each line profile are superimposed along the same axis for comparison. We see that nearly everywhere, the peak energies show only small differences but that a noticeable difference appears as we approach the endpoints *q* = |**G**_*a*, *b*_|. We therefore distinguish between the branches along the two axes by relabeling them as **q**_4, *a*_ and **q**_4, *b*_. As *q* approaches |**G**_**a,** **b**_|, the energy splitting between the **q**_4, *a*_ and **q**_4, *b*_ branches approaches ∼12 meV. This is consistent with the splitting observed in intra-unit-cell sampling of $\frac{dI}{dV}\left(r,E\right)$ data shown in Fig. 2. The ∼10 meV difference in the absolute energies of the two branches as compared to those of the two peaks in Fig. 2*J* is likely due to the fact that the measurements were acquired on two different sample surfaces.

Scattering vectors equal to **G**_*a*_ (**G**_*b*_) in *q*-space serve to connect ${\bar{X}}_{a}$ (${\bar{X}}_{b}$) and its counterpart at the opposite edge of the BZ. This suggests that the **q**_4_ branch captured in Figs. 3 and 4 results from scattering between the electron pockets around the $\bar{X}$ points. Fig. 4 *D* and *E* depict this scenario with reference to the calculated spectral function and show the appearance of **q**_4_ in the calculated QPI pattern.

## Origin of Nematicity via the Density Wave Equation

The results presented above describe symmetry breaking both in the intra-unit cell density-of-states and also in quasiparticle scattering between the pairs of electron-like pockets around opposing ${\bar{X}}_{a}$ and ${\bar{X}}_{b}$ points. We interpret these results as different manifestations of the same underlying phenomenon. The latter manifestation strongly suggests a *k*-dependent shift of the band energies, that has an opposite sign near ${\bar{X}}_{a}$ as compared to ${\bar{X}}_{b}$, and this would also give an intuitive explanation for the former manifestation: The electron bands flatten out exactly at the ${\bar{X}}_{a,b}$ points so that the last remaining scattering vectors connecting the pairs of bands at the band bottoms are exactly **q**_*a*, *b*_ = **G**_*a*, *b*_. The **q**_*a*_ and **q**_*b*_ striped patterns each appear at the energy that the respective band bottoms out. The simplest *k*-dependent energy correction that explains the above results is one with *d*-form factor.

A structural distortion is unlikely as the origin of the observed lifting of degeneracy because BaNiS_2_ is tetragonal at room temperature, and no phase transition is reported down to *T* = 2 K (22). It may be more reasonable to assume an electronic origin. We exclude a simple orbital ordering, however, because there is apparently no degeneracy of the relevant orbitals that make up the pocket around the $\bar{X}$ points (as among *d*_*xz*_ and *d*_*yz*_, for example). Moreover, the small density-of-states near *E*_F_ likely excludes mechanisms stemming from a Fermi surface instability.

For these reasons, we adopt a framework based on the DW equation, which is based on a microscopic theory beyond the mean-field theory and has previously been used to discuss *d*-form factor energy corrections emerging from many-body correlations in the context of the unconventional superconductors (31, 32, 33, 34, 35, 36, 37) as well as exotic density-wave states in other systems (38). In this scenario, broken symmetry in the charge sector may arise as a many-body correction generated by the interference of spin fluctuations.

**Fig. 5.**
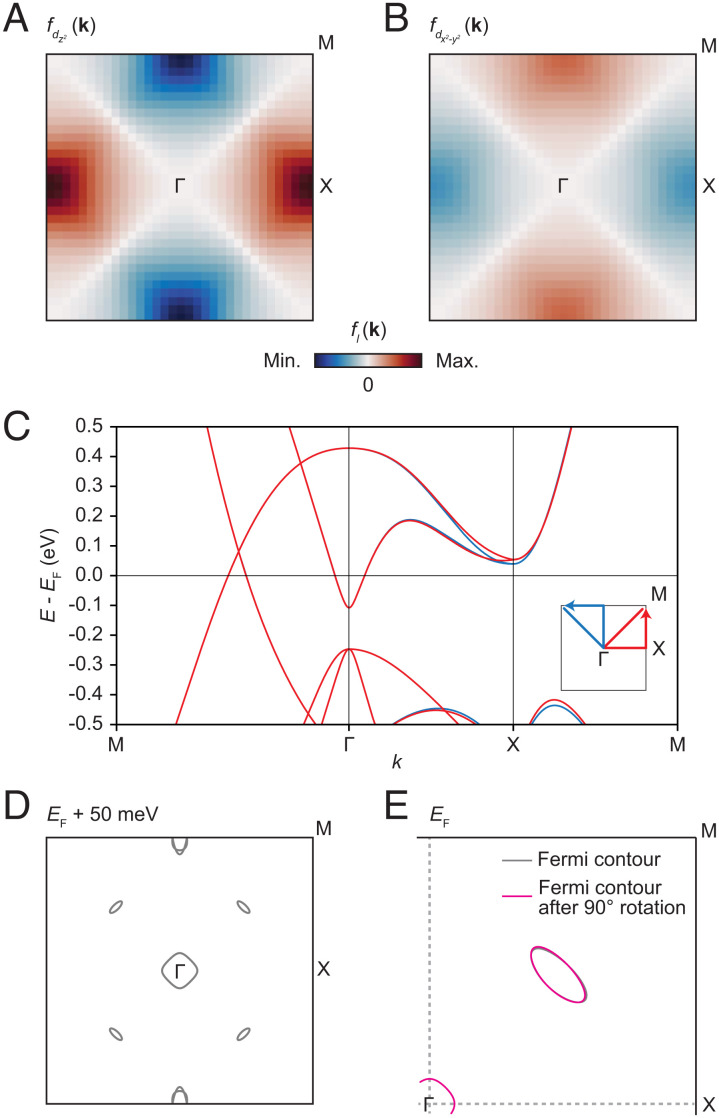
Symmetry breaking corrections via the density wave equation. (*A*) Calculated form factor *f*_*d*_*z*^2^__(**k**). (*B*) Corresponding form factor *f*_*d*_*x*^2^ − *y*^2^__(**k**). (*C*) Band structure after corrections, plotted along two paths through the BZ that capture the rotational symmetry breaking (see inset). The corrected curves are shown in red and blue, corresponding to the red and blue paths depicted in the inset. They exhibit a significant difference in energy near the X-point. (*D*) A constant-energy band contour plot at *E*_F_ + 50 meV, above the bottom of one set of electron-like pockets but below the bottom of the other. (*E*) Effect of *d*-form factor corrections on the Dirac cone. Constant energy contour in the positive quadrant of the BZ, at *E*_F_, showing the contour of a Dirac cone (gray). A contour from another Dirac cone, rotated by 90° into the positive quadrant, is also shown (magenta).

Fig. 5 shows results of numerical calculations of *d*-form factor energy corrections *f*_*l*_(**k**). The full details of the calculations are described in the Methods section. Fig. 5 *A* and *B* shows plots of the form factors calculated for the two dominant orbitals of the electron-like band near X, namely *d*_*z*^2^_ and *d*_*x*^2^ − *y*^2^_ (recall Fig. 1 *D* and *F*). The strength of each *f*_*l*_(**k**) is plotted with a common color scale. Although *f*_*d*_*z*^2^__(**k**) and *f*_*d*_*x*^2^ − *y*^2^__(**k**) are related by a sign change and therefore partially cancel each other, the former is a factor of ∼2 stronger than the latter, and a significant *d*-form factor correction remains. Note that an inverse Fourier transform of the *d*-form factor results in a *B*_1*g*_ bond-order pattern in real space, similar to that illustrated in Fig. 2*G*. Fig. 5*C* shows the resulting band structure after applying the correction to the band energies, sampled along two paths in the BZ whose ΓX segments run perpendicular to each other, as shown in the inset. The blue and red curves show a negligible difference except in the region near the X-point. This difference serves to explain the observations of rotational symmetry breaking in QPI and in high-resolution spectroscopic imaging described in the sections above. Fig. 5*D* shows the constant-energy contours of the corrected band structure at the energy of 50 meV, roughly the energy at which nematicity was observed. In Fig. 5*E*, we plot one quadrant of the BZ showing the Fermi contour of a Dirac cone and that of another Dirac cone rotated by 90° from an adjacent quadrant. This visualizes the effect of the *d*-form factor correction on the Dirac cones, which is seen to be fairly small. This is because the ΓM lines on which the Dirac cones are centered are the nodes of the form factor, as shown in Fig. 5 *A* and *B*.

Short discussions of the energy savings originating from the emergence of *B*_1*g*_ bond order and the relation between nematicity in BaNiS_2_ and magnetism in BaCoS_2_ are given in *SI Appendix*.

## Conclusion

To summarize the results of this work, we have characterized the cleaved (001) surface of BaNiS_2_ using STM, where we can access the electronic properties of the uppermost Ni square net. Through tunneling spectroscopy (including Landau level spectroscopy), we determine the approximate energy of the Dirac nodal line to be *E*_D_ ≈ 27 meV. We estimate the Fermi velocities on the inner and outer arcs of each Dirac cone, along the $\bar{\Gamma M}$ line, as *v*_*k*, inner_ = 3.09×10^5^ m s^−1^ and *v*_*k*, outer_ = −2.98×10^5^ m s^−1^, respectively. This is in fairly good agreement with previous reports (13).

High-resolution conductance imaging and QPI observations both reveal a lowering of symmetry, from the *C*_4_ symmetry expected for a material belonging to the *P*4/*nmm* space group to *C*_2_. The *C*_2_ configuration consists of a pair of striped patterns appearing at around 60 meV above *E*_F_, related to each other by a 90° rotation and ∼12 meV energy splitting, and oriented along the Ni bond chains. This symmetry-breaking phenomenon appears to respect the translational symmetries of the surface lattice but break its rotational symmetry. Hence, it constitutes an example of nematic order.

A plausible explanation for the observed nematicity is the *d*-form factor modification, *f*_*l*_(**k**), to the hopping integrals on the Ni square net, characterized within the DW equation framework as resulting from interference between spin fluctuations. Because the Dirac points lie on the nodes of *f*_*l*_(**k**), they are almost unaffected by the nematicity. An additional unusual feature of nematicity in BaNiS_2_ is that it is present even in a semimetal, having very low density-of-states at *E*_F_. This is in sharp contrast with mechanisms such as the Peierls-type instability or the band Jahn–Teller effect, which are usually promoted by a large density-of-states at *E*_F_ and a correspondingly large available energy-saving upon reconfiguration of the Fermi surface.

From the above findings, we conclude that, in BaNiS_2_, topological Dirac nodal lines and symmetry-breaking electronic correlations coexist without the former being significantly perturbed by the latter. However, looking ahead, the investigation of the BaCo_1 − *x*_Ni_*x*_S_2_ solid solution will be of interest because substitution with Co should be expected to increase the influence of electronic correlations on the electronic behavior. Ultimately, this may allow us to observe the fate of topologically protected Dirac cones as the system is driven toward a Mott transition. Doping with Co has been shown to have the effect of raising the Dirac point energy (13) and likely also raises the energy of the electron-like band around $\bar{X}$. Achieving an opposite doping effect to instead bring the electron bands down to *E*_F_ may also yield interesting behavior.

Overall, as well as adding to the understanding of the nearly ideal Dirac nodal line electronic structure of BaNiS_2_, the presented here also advance the general understanding of electronic nematic states in correlated transition metal square lattice systems.

## Materials and Methods

### STM Measurements.

Single crystals of BaNiS_2_ were synthesized as described previously (39, 40). Samples were prepared for measurement by cleaving in an ultra-high vacuum chamber (*P* ∼ 10^−10^ Torr) on a cleaving stage held at about 77 K. They were then quickly inserted into a modified Unisoku 1300 low-temperature STM system held at 1.5 K (41), equipped with a homemade head similar to that described previously (42). Scanning tips were formed by electrochemically etching tungsten wire and characterized and conditioned using a field ion microscope and mild indentation at a clean Cu(111) surface. To measure differential conductance $\frac{dI}{dV}$, a lock-in technique was used, with modulation frequency *f*_mod_ = 617.3 Hz. The modulation amplitude *V*_mod_ used for each measurement, where relevant, is specified in the respective figure caption.

### STM Data Processing.

To prepare *L*(**r**) image data for fast Fourier transformation, one of two methods was used. For all data shown in Fig. 2, a Hann window was applied before transformation. For all imaging of QPI patterns in Figs. 3 and 4, Moisan’s “periodic plus smooth image decomposition” method was used (43). This avoids the introduction by the Hann window of periodic envelope artifacts in the *q* domain (visible in *SI Appendix*, Fig. S1*B*).

### Density Functional Theory and Tight-Binding Model Calculations.

To model QPI patterns, we first performed density functional theory (DFT) calculations for bulk BaNiS_2_ using the Perdew–Burke–Ernzerhof exchange-correlation functional (44), as implemented in the WIEN2k package (45). We also performed additional calculations using a modified Becke–Johnson exchange potential (46), which is a nonlocal exchange potential that can partially account for the many-body correlation effects not treatable within DFT. These calculations are referred to as correlation-corrected DFT, or in short CC-DFT. For both sets of calculations, we sampled the bulk BZ using a 20 × 20 × 10 *k*-mesh and muffin-tin *R*_MT_ radius for all atoms such that its product with the maximum modulus of reciprocal space *K*_max._ becomes *R*_MT_*K*_max._ = 7.0. The bulk DFT and CC-DFT calculations were then down-folded into a 20-band tight-binding model using maximally localized Wannier functions (47, 48) incorporating Ni-3*d* orbitals as the projection centers. The tight-binding Hamiltonian was then used to construct a 100-layer supercell stacked along the crystalline *c*-axis, while spanning the surface BZ over a fine 256 × 256 *k*-mesh.

As the CC-DFT results more successfully reproduced observed electronic structures, most notably the electron-like pocket around the $\bar{\Gamma }$-point in photoemission measurements (13), these were adopted for the comparisons discussed in this work.

### Calculations of QPI Patterns.

With the projected wave functions of the topmost two Ni layers, calculations of QPI were carried out using the *T*-matrix formalism, in a manner similar to that described previously (49). The lifetime broadening was chosen to be 1 meV, and a localized scalar scatterer with a scattering potential of 0.1 eV in the unitary limit was employed. We performed a basis transformation from the lattice model to the continuum model using the Wannier function (50) constructed from the tight-binding wavefunctions between ±1 eV at a height of 1 nm above the Ni_A_ plane. Lock-in broadening and normalization according to the integrated density-of-states were included for direct comparison with the experimental results. Simulations were carried out for a scatterer at both Ni_A_ and Ni_B_ sites and summed to simulate a random distribution of impurities.

### Calculations of *d*-Form Factor Correction via DW Equation.

Based on the 20-band tight-binding model derived from the CC-DFT results described above, a 2-D five-band tight-binding model was constructed by ignoring interlayer hopping, ignoring spin, and moving from the “folded” to the “unfolded” BZ. In this way, the Ni_A_ and Ni_B_ sites are treated identically. This model is equivalent to one using the folded BZ if the spin–orbit interaction and interlayer hopping are both ignored. (The results are displayed in Fig. 5 after returning to the folded BZ.)

With this model as a starting point, and following previous works, the linearized DW equation (33) can then be used to characterize rotational symmetry breaking in the hopping integrals. As in previous treatments, the DW interaction includes the Maki-Thomson, Hartree, and Aslamazov-Larkin terms, the latter of which captures the effects of interference between spin fluctuations, and can contribute a *d*-form factor correction (32). The interaction is quantified by the momentum-dependent form factor *f*_**q**, *l*_(**k**), where *l* denotes a particular orbital. With **q** = 0, *f*_*l*_(**k**) can describe a uniform bond order as suggested by observation.

We set *T* = 40 meV and *J*/*U* = 0.1, where *J* is the Hund’s coupling, and *U* is the on-site Coulomb interaction.

## Supplementary Material

Appendix 01 (PDF)

## Data Availability

All study data are included in the article and/or *SI Appendix*.
