# Supplementary material for: Correlation-driven electronic nematicity in the Dirac semimetal BaNiS2
Source: Proc Natl Acad Sci U S A. 2022 Dec 2;119(49):e2212730119. doi: 10.1073/pnas.2212730119 (PMC9894198; doi:10.1073/pnas.2212730119)
Supplement: Supplementary file 1 — Appendix 01 (PDF) [file pnas.2212730119.sapp.pdf]

Supplemental Information for: Correlation-driven electronic nematicity in the Dirac semimetal $BaNiS_2$

C. J. Butler,[1,*] Y. Kohsaka,[1] Y. Yamakawa,[2] M. S. Bahramy,[3]
S. Onari,[2] H. Kontani,[2] T. Hanaguri,[1,†] and S. Shamoto[4,5]

[1] *RIKEN Center for Emergent Matter Science, 2-1 Hirosawa, Wako, Saitama 351-0198, Japan*
[2] *Department of Physics, Nagoya University, Furo-cho, Nagoya 464-8602, Japan*
[3] *Department of Physics & Astronomy, University of Manchester, Oxford Rd., Manchester M13 9PL, United Kingdom*
[4] *Neutron Science and Technology Center, Comprehensive Research Organization for Science and Society, Tokai, Ibaraki 319-1106, Japan*
[5] *Department of Physics, National Cheng Kung University, Tainan, Taiwan 70101, Republic of China*

I. FURTHER RESULTS FROM STM TOPOGRAPHY

As indicated in Fig. 1(b) of the main manuscript, cleavage can occur between adjacent BaS layers revealing (001) facets. The STM topography shown in Fig. 1(g) is repeated in Fig. S1(a) below. Its Fourier transform $\mathcal{F}[T(\mathbf{r})]$ is shown in Fig. S1(b) and the primary set of reciprocal lattice peaks represent a periodicity of 4.52 Å, close to the reported value for the surface lattice vectors $a = b = 4.43$ Å [1]. Although the Ba and apical S are uppermost at the cleaved surface, because the density-of-states at low energies is dominated by Ni orbitals, the atomic corrugation likely represents the Ni square net. Moreover, because the observed reciprocal lattice vector indicates only one Ni atom per unit cell is observed, the corrugation therefore represents specifically the uppermost Ni_A square net.

An additional topography image that includes a step between two atomic terraces is shown in Fig. S1(c), and a height profile, shown in Fig. S1(d), shows an inter-layer separation of 9 Å, close to the lattice parameter of $c = 8.89$ Å [1].

II. LANDAU LEVEL SPECTROSCOPY

Under high magnetic fields, in suitably two- or quasi-two-dimensional metals, Landau quantization can be distinguished in conductance spectra measured using STM, in which Landau levels appear as conductance peaks [2–4]. Figure S2(a) shows $\frac{dI}{dV}(E)$ spectra acquired under various magnetic fields. The magnetic field was applied approximately parallel to the c-axis. (Surface tilt was measured to be less than 1°.) Each curve represents the average over the same field of view as for Figs. 1(h) and 1(i) in the main text. The magnetic field was increased in increments of 1 T, up to 6 T, and then in increments of 0.5 T, up to the maximum field of $H = 12$ T.

A subtle change in the spectra appears in the region between $E = 0$ and 50 meV, and this is clarified upon taking the zero-field conductance curve as a baseline and subtracting it from all other curves, as shown in Fig. S2 (b). Two peaks are seen to become prominent approaching $H = 12$ T. The peak at lower energy shifts significantly with magnetic field, while the peak at higher energy appears to have only a small shift.

Knowing the energy of the zeroth Landau level of an ideal topological, massless Dirac fermion system should have no field-dependence (other than due to the Zeeman effect), we anticipate that the upper of the two peaks in Fig. S2 (b) can be assigned LL_0, and the lower of the two can then be assigned as LL_{-1}. In the more general case in which a Dirac cone may exhibit a finite mass m^*, even the energy of the zeroth Landau level can exhibit a small linear relation with magnetic field H. This is quantified within the derivation for the energy of the nth Landau level given by Fu *et al.* [4]. We follow from this derivation, but neglect the Zeeman term, because no Zeeman splitting is discernible in the $\frac{dI}{dV}(E)$ curves despite the two-fold degeneracy of the Dirac points in $BaNiS_2$. We write the resulting relation as

$$E_n = E_{\mathrm{D}} + \hbar\omega_c n + sgn(n)\sqrt{2|e|\hbar\bar{v}^2(n+\gamma)\mu_0 H + (\frac{1}{2}\hbar\omega_c)^2} \quad (1)$$

where E_{D} is the Dirac point energy, $\hbar$ is the reduced Planck constant, $\omega_c = \frac{|e|\mu_0 H}{m^* m_e}$ is the cyclotron frequency, e is the electronic charge, $\bar{v}$ is the average velocity on the contour of the band from which the Landau level forms, and μ_0 is the vacuum permeability. The phase factor γ is given by $\gamma = \frac{1}{2} - \frac{\Phi_{\mathrm{B}}}{2\pi}$ where Φ_{B} is the Berry phase accumulated around the Landau orbit. This signifies the topological character of the system and takes the value π if the orbit encloses a non-trivial Dirac node. In the case that $\gamma = 0$, the energy of the zeroth Landau level (LL_0) remains at E_{D} independent of magnetic field.

Using this framework, and adopting the assumption that $\gamma = 0$, we estimate E_{D} and m^* by fitting to the data as shown in Fig S2(c). For this procedure, after a two-point (0.5 meV) Gaussian filter is applied to smooth each curve, the Landau level peak energies are initially guessed

* christopher.butler@riken.jp
† hanaguri@riken.jp

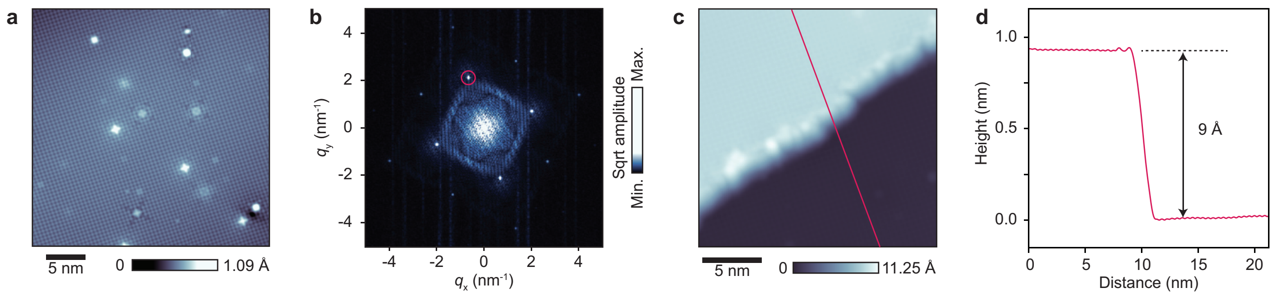

FIG. S1. **Additional details from STM topographic images.** (a) A typical constant-current STM topograph (setpoints $V = 0.1\,\mathrm{V}$, $I = 100\,\mathrm{pA}$), and (b) its Fourier transform. The peak circled in red corresponds to a reciprocal lattice vector with a norm of $0.221\,\mathrm{Å}^{-1}$ and corresponding periodicity of $4.52\,\mathrm{Å}$, close to the expected lattice parameters a and b. (c) A constant-current topograph of a step between atomic terraces ($V = 0.1\,\mathrm{V}$, $I = 10\,\mathrm{pA}$), and (d) the corresponding height profile along the red line in (c). The observed height of $9\,\mathrm{Å}$ closely matches the lattice parameter c.

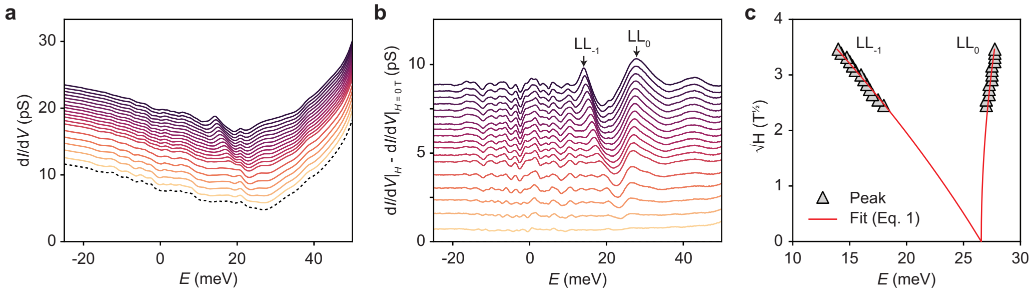

FIG. S2. **Landau level spectroscopy.** (a) $\frac{\mathrm{d}I}{\mathrm{d}V}(E)$ in the region of E_F and E_D, under magnetic field varying between 0 and 12 T. The curves are averaged over the field of view marked as a dashed square in Fig. 1(g). Each curve is offset vertically by an amount proportional to the applied magnetic field. (b) The field-dependence as represented by subtracting the zero-field curve [dashed curve in (a)] from all others. Each curve is offset vertically as in (a). Anticipating the following result, we label the peak near to E_D and the first peak below it as LL_0 and LL_{-1}, respectively. (c) Results of fitting the two labeled peaks using Eqn. 1, and using field-dependence curves from 6 to 12 T in increments of 0.5 T.

using $E_{\mathrm{peak},H} = \underset{E}{\mathrm{argmin}}\left[\frac{\mathrm{d}^2}{\mathrm{d}E^2}\left(\frac{\mathrm{d}I}{\mathrm{d}V}_H(E) - \frac{\mathrm{d}I}{\mathrm{d}V}_{H=0\mathrm{T}}(E)\right)\right]$. We also include the constraint that the fitted relations for the two Landau levels must be kept consistent with each other in that they converge upon the same value of energy at $H = 0$ T, namely E_D.

Given the above assignment of Landau level indices to the peaks, and other assumptions, the fitting estimation yields $E_\mathrm{D} \approx 26.6\,\mathrm{meV}$ and $m^* \approx 0.6$.

To summarize the key conclusions from Landau level spectroscopy results, we have determined that i) the minimum in $\frac{\mathrm{d}I}{\mathrm{d}V}(E)$ at $E - E_F = 27$ meV, seen in Fig. 1(i) of the main text, does correspond to the Dirac point energy, and also that ii) the observation of a Landau level with a weakly linear field-dependence, apparently due to finite mass, is consistent with a Berry phase of π, signifying the topologically non-trivial nature of the Dirac cones in $\mathrm{BaNiS_2}$.

III. ENERGY-DEPENDENT INTENSITIES OF BRAGG PEAKS IN FIGURE 2

Figure 2 of the main text displays intra-unit-cell sampling of the tunneling conductance $\frac{\mathrm{d}I}{\mathrm{d}V}(\mathbf{r}, E)$ in a small field-of-view, in which nematicity manifests as a difference between curves sampled at the two inequivalent bond-centered sublattices of the uppermost Ni square net. An alternative visualization of nematicity is suggested by the asymmetry between Bragg peaks in the Fourier transforms of the $L(\mathbf{r})$ images [Figs. 2(e) and 2(f) of the main text].

In Fig. S3 we shows the energy-resolved intensities of the Bragg peaks in $L_q(\mathbf{q}, E)$, for comparison with the real-space sampled $\frac{\mathrm{d}I}{\mathrm{d}V}(E)$ and $\frac{\mathrm{d}^2I}{\mathrm{d}V^2}(E)$ curves in Figs. 2(i) and 2(j) of the main text.

IV. NEMATICITY ACROSS A STEP-TERRACE MORPHOLOGY

Figure S4 shows measurements across a step-terrace morphology found at a $\mathrm{BaNiS_2}$ surface. Two steps were

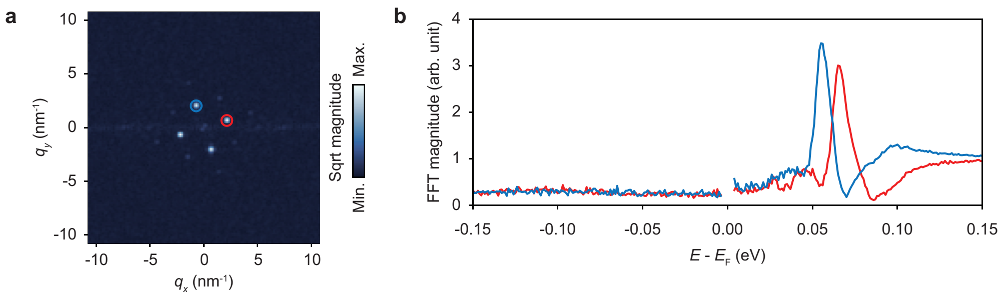

FIG. S3. **Energy-dependent intensities of Bragg peaks in Figure 2.** (a) The fast Fourier transform of the topographic image shown in Fig. 2(a) of the main text. The Fourier transform of the accompanying normalized conductance $L_q(E, \mathbf{q})$ is sampled using two masks (red and blue circles) that select the signal near each of the Bragg peaks. (The mask radius is 3 pixels, and the image size is 129×129 pixels.) (b) The intensities $L_q(E, \mathbf{q} \approx \mathbf{G}_a)$ and $L_q(E, \mathbf{q} \approx \mathbf{G}_b)$ sampled within the red and blue circles. The signal near E_F is suppressed due to noise resulting from a small denominator upon normalization.

found, separating three adjacent terraces [the lower terrace, seen at the bottom of Fig. S4(a) is the same one as at the upper terrace seen at the bottom of Fig. S4(d)]. Images of $L(\mathbf{r})$ and their Fourier transforms $L_q(\mathbf{q})$, acquired with bias separation of 10 mV, capture the presence of the nematicity described in the main text. The $L(\mathbf{r})$ and $L_q(\mathbf{q})$ images acquired on the upper and lower terraces, in the regions marked by the dashed squares in Fig. S4(a), are shown in Figs. S4(b) and S4(c), respectively. The corresponding data acquired above and below the second step, in the regions marked by dashed squares in Fig. S4(d), are shown in Figs S4(e) and S4(f), respectively.

Figures S4(c) and S4(e) represent data collected on the same terrace [as marked by the asterisk in Figs S4(a) and S3(d)], but have a different appearance due to the STM tip becoming contaminated by during the series of measurements.

Each set of images exhibits nematicity with the same orientation. This observation is consistent with two possible scenarios. First, the nematicity may be present in the bulk as well as at the surface, and have a 'ferro'-type out-of-plane correlation. Second, the nematicity in the three layers may be uncorrelated, but triggered by a global condition such as a small macroscopic sample strain. The latter scenario allows that nematicity emerges only at the surface, but is absent in the bulk.

The density-wave equation calculations presented in the latter part of the main text are based on a purely 2D system which applies to both the surface and the bulk if inter-layer hopping is neglected. The effect of three dimensionality on the nematicity is an interesting issue for future investigation.

V. FITTING OF QUASIPARTICLE INTERFERENCE SIGNALS

Here we describe the procedure for fitting to the linecut along $\mathbf{G}_a$ taken through the $L_q(\mathbf{q}, E)$ data, displayed in Fig. 3 of the main text. This leads to the acquisition of solid red and orange lines for $\mathbf{q}_2$ and $\mathbf{q}_3$ shown in Fig. 3(c) of the main text. The same procedure is also applied to the linecut data along $\mathbf{G}_b$, in order to make the comparison shown in Fig. 4 of the main text.

A model consisting of the sum of multiple Lorentzian lineshapes and a constant offset, c_q, is fitted at each point along the q path. This is written as

$$L_q^{\mathrm{fit}}(E) = \sum_{n=0}^{N} \frac{I_n(\Gamma_n/2)}{(E - E_n)^2 + (\Gamma_n/2)^2} + c_q \qquad (2)$$

Here N is the number of Lorentzian lineshapes included in the model, which is automatically determined for each q point using a peak finding algorithm [5] after some smoothing.

Figures S5(a) and S5(b) show the raw linecuts through the measured $L_q(\mathbf{q}, E)$ data, along the $\mathbf{G}_a$ and $\mathbf{G}_b$ reciprocal lattice vectors. Figures S5(c) and S5(d) show fitting results as 2D images, for comparison against the measured data. Figures S5(e) and S5(f) show the same fitting results as points with 'error bars' that signify the Lorentzian widths Γ_n. The subset of points attributed to scattering within and between the Dirac cones were chosen for subsequent linear fits. The results of these linear fits are displayed as the solid red and orange lines shown in Fig. 3(c) of the main text., and provide quantitative information about the Dirac cones, as discussed in the following section.

VI. QUANTITATIVE DESCRIPTION OF DIRAC CONES DERIVED FROM QPI RESULTS

As described in the main text, the apparent dispersion of a QPI signal has only an indirect relation to the actual dispersion of the related bands in k-space, and for this reason interpretation of QPI observations is generally not straightforward. In the present case the relation is complicated by the fact that the Dirac cones are not centered at $\mathbf{q} = 0$, and that only scattering between Dirac cones is observed, and not scattering within a single one.

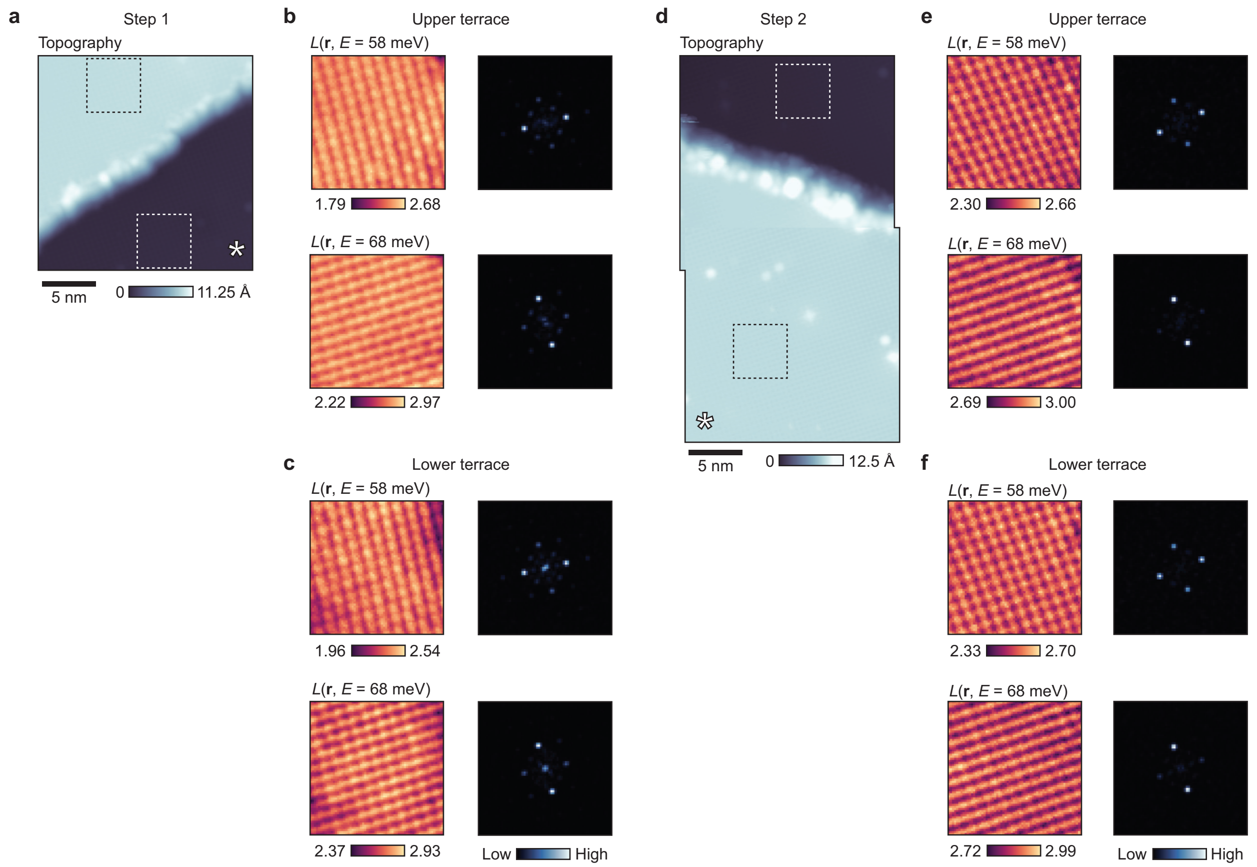

FIG. S4. **Nematicity in three adjacent terraces.** (a) Topography showing a single unit-cell high step (setpoints $V = 0.1\,\mathrm{V}$, $I = 100\,\mathrm{pA}$). (b) Images of $L(\mathbf{r})$ and their Fourier transforms $L_q(\mathbf{q})$ at $E = 58\,\mathrm{mV}$ and $68\,\mathrm{mV}$. (c) The corresponding images acquired on the lower terrace. (d) Topography showing another single unit-cell high step. The higher terrace (at the bottom of the image) is the same as that shown in the lower right of (a), as indicated by the asterisk symbols. (e) and (f) The corresponding $L(\mathbf{r})$ and $L_q(\mathbf{q})$ images at $E = 58\,\mathrm{mV}$ and $68\,\mathrm{mV}$. Here nematicity manifests as a differing intensity between the $L_q(\mathbf{q})$ peaks corresponding to the reciprocal lattice vectors $\mathbf{G}_a$ and $\mathbf{G}_b$. The orientation of the striped patterns is the same for all three terraces.

Nevertheless below we draw a comparison between the observed and calculated features in q-space, and also a k-space-to-q-space comparison of the calculation results, specifically for the QPI signals $\mathbf{q}_2$ and $\mathbf{q}_3$ shown in Fig. 3 of the main text. From this we discuss whether the Dirac point energy can be inferred from QPI observations, and also derive estimates of the Dirac cone velocities parallel to the $\overline{\Gamma\mathrm{M}}$ line.

First, if the observed scattering branches $\mathbf{q}_2$ and $\mathbf{q}_3$ result from scattering between adjacent Dirac cones as depicted in Fig. 3(d) of the main text, they intersect at the energy where each of the Dirac cones vanishes to a point, so that the shortest and longest possible vectors connecting their contours (namely $\mathbf{q}_2$ and $\mathbf{q}_3$) become identical. The energy at which they intersect ($E = 101\,\mathrm{meV}$) would then seem to be the Dirac point energy. However, we find that this is not true. The calculated Dirac point energy is in fact about $60\,\mathrm{meV}$ [see Fig. 1(d) in the main text], meaning the extrapolated point of intersection gives an over-estimate, perhaps due to a finite curvature as the band nears the Dirac point. This indicates that an extrapolation from the branches $\mathbf{q}_2$ and $\mathbf{q}_3$ in the observation (which would yield $E_{\mathrm{Dirac}} \approx 47\,\mathrm{meV}$) would similarly over-estimate the actual Dirac point energy. For this purpose we fall back on the minimum in the $\frac{\mathrm{d}I}{\mathrm{d}V}(E)$ curve [Fig. 1(i) of the main text], and also Landau level spectroscopy above, to determine the Dirac point energy.

As described above, the point of intersection of $\mathbf{q}_2$ and $\mathbf{q}_3$ represents the q-vector connecting two adjacent Dirac points, which we may call $\mathbf{q}_{\mathrm{D-D}}$. The assumption that each Dirac point lies on a $\overline{\Gamma\mathrm{M}}$ line in k-space leads to $|\mathbf{q}_{\mathrm{D-D}}|^2 = 2\,|\mathbf{k}_{\mathrm{D}}|^2$ where $\mathbf{k}_{\mathrm{D}}$, the wavevector of the Dirac point, is directed from the origin along the $\overline{\Gamma\mathrm{M}}$ line. This results in $|\mathbf{k}_{\mathrm{D}}| \approx 0.884\,\mathrm{nm}^{-1}$.

An approximate value for the Dirac cones' Fermi velocity $v_{\mathrm{F}} = \frac{\mathrm{d}E_{\mathrm{F}}}{\mathrm{d}\mathbf{k}}$ along a particular axis can be inferred from the apparent velocity $v_{q,2,3}$ of the scattering branches $\mathbf{q}_2$ and $\mathbf{q}_3$. We start from the assumption that v_q for a generic scattering branch always relates to the velocity v_k of a chosen point on the band by a proportionality constant that captures geometric details of the scattering vectors allowed within the band's structure, i.e. $v_q = c v_k$.

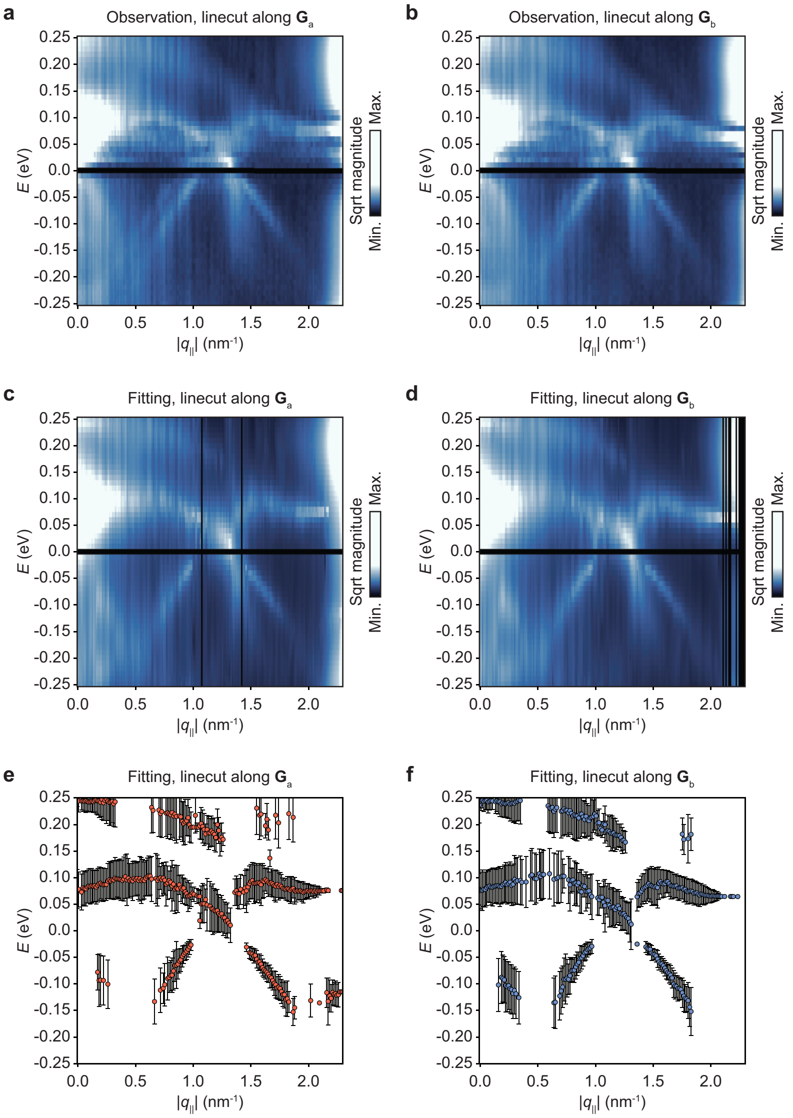

FIG. S5. **Fitting to $L_q(\mathbf{q}, E)$ data.** (a) and (b) Linecuts through measured $L_q(\mathbf{q}, E)$ data, along the $\mathbf{G}_a$ and $\mathbf{G}_b$ reciprocal lattice vectors. (c) and (d) Corresponding images composed of the fitting results at each q point, with a fitting model described by Eq. 2. Where the fitting procedure failed to converge, a vertical black line is shown. (e) and (f) The same fitting results displayed as points and 'error bars' (which in this case denote the Lorentzian broadening Γ). These plots are superimposed together in Fig. 4(c) of the main text.

(As an example, $v_q = \frac{1}{2}v_k$ in the extremely simple case of scattering across the diameter of an isotropic Dirac cone).

Next, we compare the calculated and observed velocities $v_q^{\text{obs.}}$ and $v_q^{\text{calc.}}$ for each branch [see Figs. 3(c) and 3(f) of the main text]. These velocities are related by a factor we call the renormalization factor R, such that $v_q^{\text{obs.}} = Rv_q^{\text{calc.}}$. Since in both calculation and in reality, $v_q = cv_k$ with the same constant c (determined only by geometry), we also posit that the real band velocity is $v_k = Rv_k^{\text{calc.}}$. In this way a renormalization of the k-space band structure can reasonably be inferred from the apparent renormalization of q-space structures.

The resulting calculated apparent velocities [see Fig. 3(f) of the main text] are $v_{q_2}^{\text{calc.}} = 1.41 \times 10^5\,\text{m}\,\text{s}^{-1}$ and $v_{q_3}^{\text{calc.}} = -1.18 \times 10^5\,\text{m}\,\text{s}^{-1}$, respectively. Comparing these with the experimental values leads to $R = 0.58$ for both inner and outer branches $\mathbf{q}_2$ and $\mathbf{q}_3$.

Using the same method of fitting as shown in Fig. S4, but for the surface spectral function shown in Fig. 1(d) of the main text, gives velocities for the inner and outer arcs of the Dirac cone, along the $\overline{\Gamma\text{M}}$ line, of $v_{k,\text{inner}}^{\text{calc.}} = 5.32 \times 10^5\,\text{m}\,\text{s}^{-1}$, and $v_{k,\text{outer}}^{\text{calc.}} = -5.14 \times 10^5\,\text{m}\,\text{s}^{-1}$, respectively. Finally, using $R = 0.58$ allows us to infer that the actual band velocities in the sample are $v_{k,\text{inner}} = 3.09 \times 10^5\,\text{m}\,\text{s}^{-1}$ and $v_{k,\text{outer}} = -2.98 \times 10^5\,\text{m}\,\text{s}^{-1}$. As a comparison, from recently reported angle-resolved photoemission spectroscopy results, we estimate corre-

sponding absolute velocities of $|v_{k,\text{inner}}| \sim |v_{k,\text{outer}}| \approx 2 \times 10^5\,\text{m}\,\text{s}^{-1}$ [6].

The band velocities along the $\overline{\Gamma\text{M}}$ line are higher than the average Fermi velocity as determined through Landau level spectroscopy above. This is partly because the Dirac cones are elliptical, having the highest velocity along $\overline{\Gamma\text{M}}$ and the lowest velocity perpendicular to this line. It may also be because the Fermi contour generally has a lower velocity as the Dirac cone has some curvature, becoming lower in velocity near E_D. The estimate of velocities based on QPI dispersions relies on energies between -50 and $-100\,\text{meV}$, where the cone has steeper and more linear dispersion.

VII. ORIGIN OF ENERGY SAVINGS DUE TO NEMATIC ORDER

Here, we discuss why an energy gain results from the present finite-energy nematic order. Although the Dirac nodes are almost unchanged by the B_{2g} form factor, the band dispersion along the antinodal direction is significantly modified. First, we discuss the change in the free-particle energy due to the form factor $f_l(\boldsymbol{k}) = f \times \bar{f}_l(\boldsymbol{k})$, where $\bar{f}_l(\boldsymbol{k})$ is the normalized form factor ($\max_{l,\boldsymbol{k}} |\bar{f}_l(\boldsymbol{k})| = 1$). In the hole representation, it is given by

$$\Delta E^f_\text{free} \equiv E^f_\text{free} - E^{f=0}_\text{free} \approx af^2 + bf^4, \tag{3}$$

$$E^f_\text{free} = \frac{1}{N}\sum_{n,\boldsymbol{k}}(1 - f(\epsilon^f_{n,\boldsymbol{k}}))(-\epsilon^f_{n,\boldsymbol{k}}), \tag{4}$$

where $f(\epsilon)$ is the Fermi distribution function, $\epsilon^f_{n,\boldsymbol{k}}$ is the n-th band dispersion with the form factor. We perform the numerical study at $T \sim 0.01$, where Eq. (3) is very close to the free energy because TS is small. In the present model, the relations $a < 0$ and $b > 0$ hold, and therefore $\Delta E^f_\text{free} < 0$ at a finite f. This energy gain mainly originates from the electron-like dispersion around the X points (Fig. 5). In fact, the bottom of this dispersion ($E_0 \approx 0.05$ eV) is given as $E_{X_a(X_b)} \approx E_0 + (-)cf + df^2$, where $c \approx 1$ and $d > 0$. In fact, $d \approx (E_0 - E'_0)^{-1}$ due to the second-order perturbation theory, and $E'_0 \approx -0.5$ eV is the energy of the nearest valence band at the X points. Thus, the negative a in Eq. (3) is robustly obtained in the present band-structure.

Next, we discuss the total energy with the electron correlation, ΔE^f. According to Ref. [7], it is simply given as

$$\Delta E^f \approx a' f^2 + bf^4, \tag{5}$$

where $a' = a(1 - 1/\lambda)$, and λ is the eigenvalue of the DW equation. Here, $\lambda > 1 (< 1)$ below (above) T_nem. Because $a < 0$ in $BaNiS_2$, a' in Eq. (5) is negative below T_nem, and the order parameter is given as $|f| = \sqrt{-a'/2b}$. According to Ref. [7], the specific heat jump at T_nem is $\Delta C/T \sim \alpha N(0)$ with $\alpha = (-\dot{\lambda}T)_{T_\text{nem}}$, where $\dot{\lambda} \equiv d\lambda/dT$. In the BCS superconductor, $\alpha = 1$. In contrast, $\alpha \ll 1$ is expected in the case of the nematic transition at $T = T_\text{nem}$ [7].

VIII. TEMPERATURE DEPENDENCE

A natural next step following on from these results is to experimentally characterize the temperature dependence of the nematic order. The energy splitting of $\sim$12 meV corresponds to a temperature of $\sim$140 K, naïvely the minimum possible transition temperature for the nematic phase. Unfortunately, tunneling spectroscopy observations suffer from an energy broadening that rises faster than $k_\text{B}T$ (by a factor of 3.5), meaning that observations similar to those presented in this work probably become impractical before reaching the temperature required to observe the potential melting of the nematic phase. This means that further investigations using other tools may be necessary to characterize its temperature dependent behavior.

IX. RELATION BETWEEN NEMATICITY IN $BaNiS_2$ AND MAGNETISM IN $BaCoS_2$

The antiferromagnetic phase of $BaCoS_2$ can be thought of as an arrangement of collinear spin chains aligned along one of the Ni_A-Ni_B axes and antiferromagnetically coupled to each other, or equivalently, a spin density wave with a wavevector $Q_\text{SDW} = (\pi, \pi)$ [8]. Likewise, in $BaNiS_2$, the magnetic nesting vector is $Q = (\pi, \pi)$. In the spin-nematic scenario, the director of nematicity is always aligned with the axis of the expected spin stripe order, which would lead to a director of nematicity oriented parallel to the Ni_A-Ni_B axis, at 45° to the pattern observed in Fig. 2 of the main text. In contrast, in the present spin-fluctuation interference scenario, there is no such constraint on the director of nematicity, allowing the configuration as observed in Fig. 2 of the main text, with $Q = (0, \pi)$ and stripes parallel to Ni_A-Ni_A.

[1] I. E. Grey and H. Steinfink *Crystal structure and properties of barium nickel sulfide, a square-pyramidal nickel(II) compound.* J. Am. Chem. Soc. **92**, 17, 5093–5095 (1970). https://doi.org/10.1021/ja00720a015

[2] P. Cheng, C. Song, T. Zhang, Y. Zhang, Y. Wang, J.-F. Jia, J. Wang, Y. Wang, B.-F. Zhu, X. Chen, X. Ma, K. He, L. Wang, X. Dai, Z. Fang, X. Xie, X.-L. Qi, C.-X. Liu, S.-C. Zhang, and Q.-K. Xue, *Landau Quantization of Topological Surface States in* Bi_2Se_3. Phys. Rev. Lett. **105**, 076801 (2010). https://doi.org/10.1103/PhysRevLett.105.076801

[3] T. Hanaguri, K. Igarashi, M. Kawamura, H. Takagi, and T. Sasagawa, *Momentum-resolved Landau-level spectroscopy of Dirac surface state in* Bi_2Se_3. Phys. Rev. B **82**, 081305(R) (2010). https://doi.org/10.1103/PhysRevB.82.081305

[4] Y.-S. Fu, T. Hanaguri, K. Igarashi, M. Kawamura, M. S. Bahramy, and T. Sasagawa, *Observation of Zeeman effect in topological surface state with distinct material dependence.* Nat. Commun. **7**, 10829 (2016). https://doi.org/10.1038/ncomms10829

[5] https://docs.scipy.org/doc/scipy/reference/generated/scipy.signal.find_peaks.html

[6] N. Nilforoushan, M. Casula, A. Amaricci, M. Caputo, J. Caillaux, L. Khalil, E. Papalazarou, P. Simon, L. Perfetti, I. Vobornik, P. K. Das, J. Fujii, A. Barinov, D. Santos-Cottin, Y. Klein, M. Fabrizio, A. Gauzzi, and M. Marsi, *Moving Dirac nodes by chemical substitution.* Proc. Natl. Acad. Sci. **118**, e2108617118 (2021). https://doi.org/10.1073/pnas.2108617118

[7] R. Tazai, S. Matsubara, Y. Yamakawa, S. Onari, H. Kontani, *A Rigorous Formalism of Unconventional Symmetry Breaking in Fermi Liquid Theory and Its Application to Nematicity in FeSe.* arXiv:2205.02280 (2022). https://doi.org/10.48550/arXiv.2205.02280

[8] D. Mandrus, J. L. Sarrao, B. C. Chakoumakos, J. A. Fernandez-Baca, S. E. Nagler, and B. C. Sales, *Magnetism in* $BaCoS_2$. J. Appl. Phys. **81**, 4620 (1997). https://doi.org/10.1063/1.365182
